# A sensitive electrochemical sensor for *in vitro* detection of parathyroid hormone based on a MoS_2_-graphene composite

**DOI:** 10.1038/srep34587

**Published:** 2016-10-03

**Authors:** Hyeong-U Kim, Hye Youn Kim, Atul Kulkarni, Chisung Ahn, Yinhua Jin, Yeongseok Kim, Kook-Nyung Lee, Min-Ho Lee, Taesung Kim

**Affiliations:** 1 SKKU Advanced Institute of Nano Technology (SAINT), Sungkyunkwan University, Suwon, Gyeonggi-do, Republic of Korea; 2 Korea Electronics Technology Institute, Seongnam, Gyeonggi-do, Republic of Korea; 3 Mechanical Engineering, Sungkyunkwan University, Suwon, Gyeonggi-do, Republic of Korea

## Abstract

This paper reports a biosensor based on a MoS_2_-graphene (MG) composite that can measure the parathyroid hormone (PTH) concentration in serum samples from patients. The interaction between PTH and MG was analysed *via* an electrochemical sensing technique. The MG was functionalized using l-cysteine. Following this, PTH could be covalently immobilized on the MG sensing electrode. The properties of MG were evaluated using scanning electron microscopy, high-resolution transmission electron microscopy, X-ray diffraction, Raman spectroscopy, X-ray photoelectron spectroscopy, and Fourier transform infrared spectrometry. Following optimization of immobilized materials—such as MG, PTH, and alkaline phosphatase (ALP)—the performance of the MG sensor was investigated *via* cyclic voltammetry, to assess its linearity, repeatability, and reproducibility. Electrochemical impedance spectroscopy was performed on graphene oxide (GO) and MG-modified electrodes to confirm the capture of a monoclonal antibody (MAb) targeting PTH. Furthermore, the ALP-PTH-MG sensor exhibits a linear response towards PTH from artificial serum over a range of 1–50 pg mL^−1^. Moreover, patient sera (n = 30) were evaluated using the ALP-PTH-MG sensor and compared using standard equipment (Roche E 170). The *P*-value is less than 0.01 when evaluated with a *t*-test using Welch’s correction. This implies that the fabricated sensor can be deployed for medical diagnosis.

Graphene (Gr) is a material composed of a single layer of densely packed carbon atoms. Its advantageous physical and chemical properties, such as high surface area, high electrical conductivity, chemical tolerance, and mechanical strength, have stimulated considerable research interest^1^,^2^,^3^,^4^. The two-dimensional (2D) planar structure of Gr affords various platforms for hybridization with different materials^5^,^6^,^7^,^8^. There can be a strongly synergistic interaction between Gr and its composite components, which can lead to enhanced catalytic activity and stability^9^. Various nanomaterials such as platinum nanoparticles with multi-walled carbon nanotubes^10^, graphene^11^ and graphene oxide^12^ have been introduced due to their ability for facilitating electron transfer of redox mediators, which leads to sensitive electrochemical aptasensors. Transition-metal dichalcogenides (TMDs) are atomically thin semiconductors of the type MX_2_, where M is a transition metal (Mo, W) and X is a chalcogen (S, Se, or Te). TMDs have attracted a great deal of interest because of their wide range of electronic, optical, mechanical, chemical and thermal properties. Molybdenum disulfide (MoS_2_), in particular, has a tunable band gap between 1.29 and 1.90 eV, depending on the number of layers^13^. Thus, MoS_2_ finds many uses: for example, in electrochemical devices, hydrogen storage, and capacitors. It is also an intercalation host and a catalyst of hydrogen evolution reactions^14^,^15^,^16^. In our previous work, MoS_2_ was successfully synthesized below 150 °C, for flexible device applications on Si/SiO_2_^17^ and polyimide (PI) substrates^18^, using plasma-enhanced chemical vapor deposition. We adopted that same technique for the direct synthesis of MoS_2_ on an Au electrode; then, we implemented a biosensor for hydrogen peroxide (H_2_O_2_) using conjugated horseradish peroxidase (HRP)-IgG-MoS_2_, which is sensitive over the range of 0–20 ng mL^−1 ^19. These results demonstrate the biocompatibility of MoS_2_ and the feasibility of immobilizing enzymes such as HRP on its surface. Consequently, sensitive biosensors might be developed by exploiting the advantageous properties MoS_2_-Gr (MG) composites. Recently, an MG composite has been successfully synthesized using either a hydrothermal^20^ or hybrid microwave annealing method^21^, and the results have been encouraging: the electron-conductivity, which is important for electrochemical applications, is improved in the MG composite compared to that of MoS_2_ on its own. A layered MG composite has been synthesized with l-cysteine and used to monitor electrochemical reactions^20^. The multifunctional groups (such as -SH, -NH_2_ and COO-) within l-cysteine facilitate the alteration/functionalization of biomolecules, hormones, proteins, and nucleic acids onto the surface of MG. MG could be used in bio-nano-composites, hydrogels, or nanomaterials containing biopolymers^22^. Moreover, MG can aid in the preservation of protein/enzyme redox activity. Thus, MG is a promising candidate material for future biosensors and electrochemical sensors.

Osteoporosis is a frequently encountered extra-articular manifestation of rheumatoid arthritis (RA). Reduction of bone mass is associated with an increased risk of RA. Parathyroid hormone (PTH) is the most important endocrine regulator of the concentration of calcium (Ca^2+^) and phosphorus in extracellular fluid, and its concentration can act as a measure of osteoporosis and parathyroid disorders. Moreover, PTH regulates calcium and phosphate metabolism in bone, kidney, and intestinal tissue, as well as in extracellular fluids^23^ Even small changes in the PTH-level within serum can provide an indication of parathyroid gland disease, such as hyper- or hypoparathyroidism. Hyperparathyroidism refers to overactivity of the parathyroid glands, which results in excessive production of PTH. Conversely, hypoparathyroidism refers to an insufficient production of PTH. Consequently, there is an opportunity to develop a highly sensitive PTH sensor, addressing an unmet need, using the nanomaterials described above. Current methods for determining the concentration of PTH include chemiluminescence^24^, electro-chemiluminescence, actuation of giant magneto-resistive particles^25^, and time-resolved fluorescence^26^. Overall, we believe that the electrochemical route is the most promising technology^27^ for detection of PTH using MoS_2_ and Gr.

The objectives of present work were: (i) To prepare a MG composite material; (ii) To immobilize (alkaline phosphatase (ALP)/HRP)-IgG on an MG-modified sensing electrode and to investigate its electrochemical performance *via* cyclic voltammetry (CV); thereby assessing enzyme sensitivity; (iii) To further utilize the enzyme-linked PTH antibody to detect the PTH (antigen) concentration in artificial sera, and also to apply the fabricated sensor in true patient samples (iv) To evaluate those by using an electrochemical immunoassay (ECIA) device.

## Results and Discussion

### MG composite

**Figure 1:**
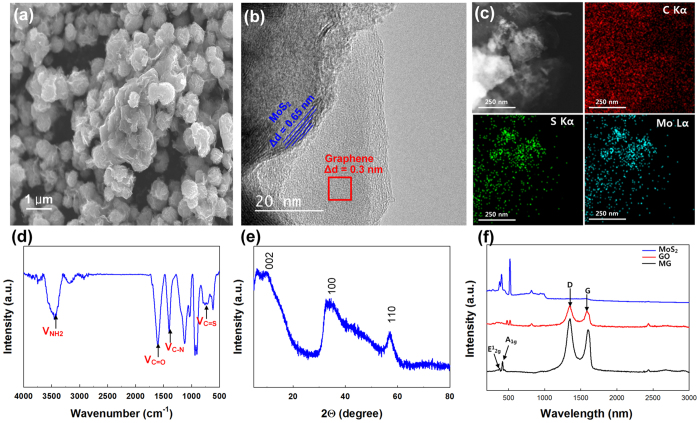
Characterization of the MG composite. (**a**) SEM image. (**b**) HR-TEM image. (**c**) TEM images and elemental mapping images obtained using EELS. (**d**) FT-IR spectra. (**e**) XRD patterns. (**f**) Raman spectra of MG, GO and MoS_2_.

The synthesized MG composite was evaluated using scanning electron microscopy (SEM), high-resolution transmission electron microscopy (HR-TEM) with element mapping, Fourier transform infrared spectrometry (FT-IR), X-ray diffraction (XRD) and Raman spectroscopy (Fig. 1). SEM confirmed that the MG composite was formed successfully by Gr self-assembly during the hydrothermal process (Fig. 1a). During the hydrothermal process graphene oxide (GO) was reduced to Gr, and coalesced particles of the MG composite were formed with a composite particle size of 1 μm^28^. The coalesced particles of the MG composite formed spheres; they had a large surface area, which might lead to efficient interactions with analytes, or the formation of a conducting network, facilitating rapid electronic transport during electrical measurements. The layered structure of MoS_2_ was visible under HR-TEM (Fig. 1b), where ‘few-layer MoS_2_’ was interconnected with Gr. Distinct differences between MoS_2_ and Gr were observed, which were confirmed by the interlayer distance measurements (0.65 nm for MoS_2_; 0.3 nm for Gr). From electron energy loss spectroscopy (EELS) of HR-TEM images (Fig. 1c), we could confirm the elemental mapping of carbon, sulphur and molybdenum, which is consistent throughout the material. From these observations, we concluded that the Gr sheet was decorated uniformly with MoS_2_ nanocrystals. The Mo precursor (Na_2_MoO_4_ · H_2_O) was mixed with l-cysteine (C_3_H_7_NO_2_S) and GO during hydrothermal synthesis; therefore, a C=S (723 cm^−1^) stretching peak and a C-N (1515 cm^−1^) peak are visible in the FT-IR spectra (Fig. 1d). Moreover, NH_2_ stretching peaks are also present, indicated by amine band I (1652 cm^−1^) and amine band II (1540 cm^−1^)^29^. A stretching peak for C=O (1611 cm^−1^) is present owing to presence of GO. Of particular interest is l-cysteine, owing to its multifunctional groups (-SH, -NH_2_ and COO-)^30^, which can be used for the conjugation of metallic ions or other functional groups. Here, l-cysteine acted as a reducing agent and a sulphur donor during the hydrothermal process, releasing H_2_S as a sulphide source. It reduced the MoO_4_^2−^ precursors from Na_2_MoO_4_ · H_2_O to MoS_2_, and reduced GO to graphene. The synthetic mechanism for MoS_2_ can be expressed as depicted in equations (1 and 2)^31^:


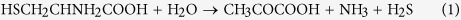



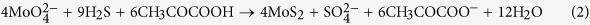


XRD analysis revealed that MoS_2_-Gr exhibits three diffraction peaks (Fig. 1e). The primary (002) diffraction peak appeared at 14.5°; however, the intensity of the peak was not strong. We attributed the poor crystallinity of MoS_2_ to the incorporation of Gr, which may have inhibited the growth of layered MoS_2_ during the hydrothermal process. Additionally, the MG composite did not exhibit a Gr (002) peak at 22°, because the Gr nanosheet was unable to form stacks during the hydrothermal process, or during annealing^32^. Raman spectroscopy has been used extensively to identify MoS_2_^13^ and Gr. Raman spectra from the MG composite demonstrated the presence of both GO and MoS_2_ (Fig. 1f). MoS_2_ has two dominant peaks, at 380 and 405 cm^−1^, corresponding to E^1^_2g_ and A_1g_ modes, respectively; MG produces identical peaks. Similarly, GO and MG have another two Raman peaks, at 1346 and 1590 cm^−1^, which are related to the D band and the G band. In general, the D band is attributed to defects and disorder in the hexagonal graphitic layers, while the G band is ascribed to the vibration of *sp*^2^ carbon atoms in a 2D hexagonal lattice. The relative intensity ratio (I_D_/I_G_) provides an indication of graphene quality. The calculated I_D_/I_G_ value of MG is increased relative to that of GO, because the MG contains some defects and portions of disordered structure resulting from hydrothermal synthesis^33^. Additionally, the presence of MoS_2_ and GO was confirmed by XPS (Fig. S1). The high resolution XPS scans revealed: (i) Peaks centred at 229.5 and 232.4 eV attributable to Mo 3*d*_5/2_ and Mo 3*d*_3/2_. (ii) Two merged peaks at 163.4 and 164.6 eV, arising from the 2*p* subshell of S, which confirms the presence of MoS_2_^34^. (iii) Peaks for C 1*s* at 284.5, 286.6 and 288.5 eV, which specify three types of carbon bonds, namely C-C, C-OH and C-O (epoxy/alkoxy), respectively.

The zeta potential is the electrokinetic potential at the interface between adsorbed surfactant molecular ions and the diffuse region of mobile counter-ions. To explain the excellent aqueous stability, the zeta potential of the MoS_2_-Gr aqueous dispersions was measured. The zeta potential of these dispersions had a negative charge of −35.25 mV (Fig. S2). This value is well above the accepted threshold value for a stable colloid (−25 mV)^35^.

### Electrochemical activity of MG

**Figure 2:**
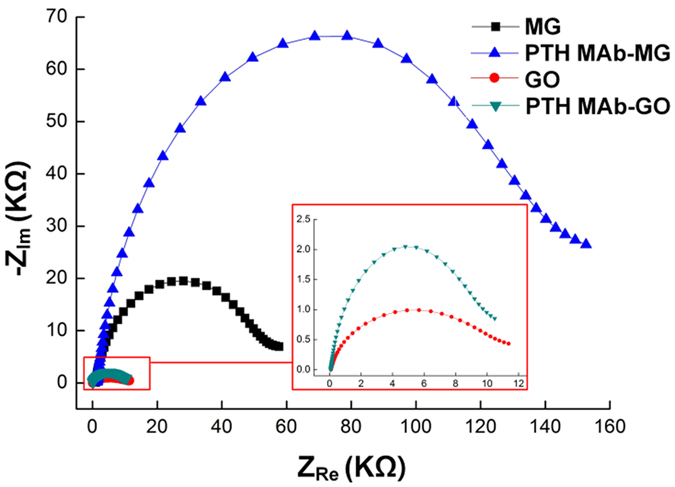
Electrochemical impedance spectra of MG- and GO-modified Au electrodes, with or without PTH MAb, in 1 mM AAP buffer. (Inset) Enlarged impedance spectra for the GO-modified Au electrode.

Electrochemical immunoassays were performed using three electrodes: a Pt wire as the auxiliary electrode, an Ag/AgCl electrode (saturated KCl) as the reference, and the MG- and GO-modified Au electrode as the working electrode. The modified electrodes were characterized by CV and by electrochemical impedance spectroscopy (EIS), which is very sensitive to surface modification. In general, EIS consists of resistive and capacitive elements besides the Warburg element and is a powerful method for analysing the complex electrical resistance of materials. In this measurement, C_d_ refers to the double layer capacitance of the system, R_ct_ refers the charge transfer resistance, Z_w_ refers to the Warburg impedance, and R_s_ refers to resistance of the electrolyte, as described in the inset of Fig. 2. The EIS spectra for the PTH MAb immobilization on the GO-modified Au electrode (PTH MAb-GO), and the MG-modified Au electrode (PTH MAb-MG), were evaluated (Fig. 2). Both modified electrodes were compared, with/without PTH MAb, in buffer (1 mM ascorbic acid-2-phosphate; AAP), at a scan rate of 100 mV s^−1^. When the modified electrodes interacted with PTH MAb, the charge transfer resistance (R_ct_) was found to undergo a pronounced increase, owing to covalent immobilization of PTH MAb on the GO and MG surfaces, which can enhance the electrical insulation.

The electrocatalytic activity was evaluated for the enzymes (HRP and ALP) immobilized by IgG on modified MG. We conjugated the IgG-linked enzymes to the MG electrode *via* the drop casting method. CV profiles were obtained with/without MG (Fig. S3a,b), using ALP-IgG (AI)/HRP-IgG (HI) in PBSB, which consisted of phosphate-buffered saline (PBS) together with 1% (w/v) bovine serum albumin (BSA). In each case, the oxidation and reduction currents increased for the MG sensor. The HRP-IgG-MoS_2_-Gr (HI-MG) sensor was oxidized, triggering the oxidation of hydroquinone (HQ) to benzoquinone (BQ), which was subsequently reduced, yielding a current response^36^. Similarly, the ALP-IgG-MoS_2_-Gr (AI-MG) sensor triggered the oxidation of ascorbic acid (AA) to AAP. AAP is novel, alternative substrate for the ALP assay, and it has been compared with *p*-aminophenyl phosphate, which is widely used for electrochemical methods in enzyme immunoassays. The procedure of the enzymatic hydrolysis reaction is described in equation (3)^37^:


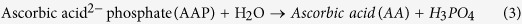


The oxidation peak was observed at 0.62 V and 0.55 V, for HI-MG and AI-MG, respectively (Fig. S3a,b). The anodic current behaviour with MG was similar to that obtained without MG; however, the current level of the AI-MG sensor was ~1.3-fold greater than that of the AI sensor without MG (Fig. S3a). Since the MG composite enhanced the binding of analytes, the current level resulting from nonspecific binding was greater for the AI-MG and the Au electrode than that for the Au electrode with AI alone. Moreover, the chemical stability of MG was high, which facilitated the conjugation of AI to the Au electrode. Similarly, the current level of the HI-MG sensor was about 111% greater than that of the HI sensor without MG (Fig. S3b). The anodic currents obtained with the MG-treated electrodes were much higher than those obtained with untreated Au electrodes.

### ALP-PTH-MoS_2_-Gr (AP-MG) sensor response

**Figure 3:**
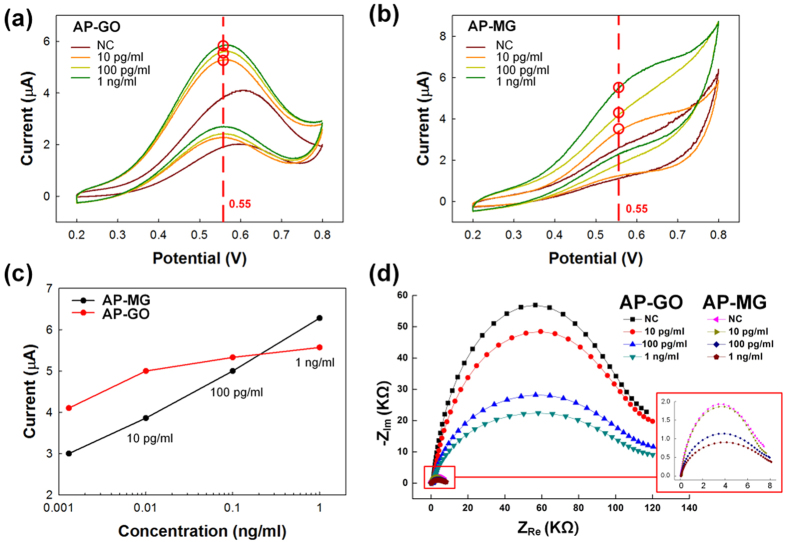
Cyclic voltammograms for (**a**) AP-GO, and (**b**) AP-MG sensors, following treatment with sandwich ELISA for 10 pg/mL, 100 pg/mL and 1 ng/mL PTH concentrations. The sensor was immersed in a 1 mM AAP solution at a scan rate of 100 mV/s. (**c**) Comparison of PTH concentration based on the oxidation peaks of the AP-MG and AP-GO sensors. (**d**) Electrochemical impedance spectra obtained using AP-GO and AP-MG sensors, at different PTH concentrations, using ALP in 1 mM AAP.

Biosensors are routinely characterized by assessing their linear range, reproducibility, and repeatability. In this study, CV was successfully used to determine the amount of PTH, as well as with ALP, in analytes using GO- (AP-GO) and MG- (AP-MG) modified electrodes. For all measurements, the different concentrations of PTH, with ALP, were compared with a negative control (NC). The role of ALP enzyme is to conjugate antibodies and AAP, where AAP used as a substrate. The enzymatic reaction between ALP and AAP results in AA as depicted in Fig. S4. The redox reaction was verified *via* CV for both GO- and MG-modified Au electrodes (Fig. 3a,b); the oxidation peak was observed at 0.55 eV. Notably, the oxidation peaks obtained from CV measurements increase linearly with increasing PTH concentrations (Fig. 3c). The increase in the peak current upon addition of PTH is attributed to the PTH MAb that was immobilized on both the GO- and MG-modified electrodes. The MG-modified electrode shows a linear variation as the concentration of PTH is changed, unlike the GO-modified electrode. Hence, the MG-modified electrodes were used for further studies. Nyquist plots were obtained for the modified electrodes at different PTH concentrations, with ALP in AAP substrate (Fig. 3d). Increasing the PTH concentration led to a decrease in the semi-circle diameter in the Nyquist plot. Increases in the concentration of PTH can, in turn, increase conjugation sites for ALP, and the ALP provides redox activity using the AAP substrate. In our study, redox reactions are initiated and the oxidation peak current is increased, due to enzymatic reactions with the ALP substrate—unlike other electrochemical sensors. Consequently, a PTH concentration-dependent decrease was seen in the R_ct_ value.

**Figure 4:**
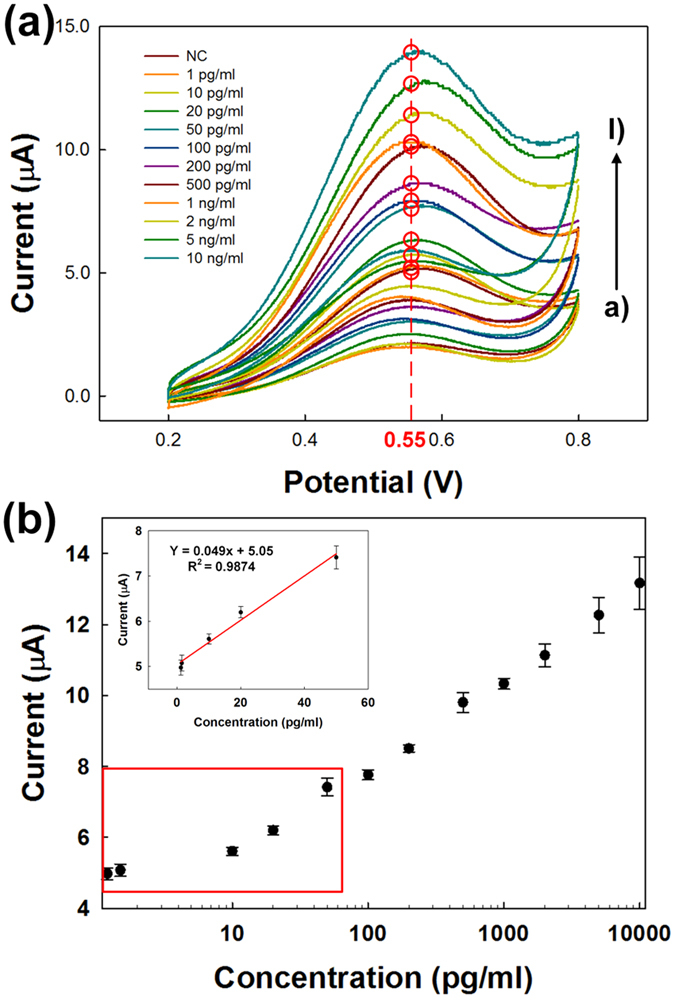
Calibration curve of the AP-MG sensor for quantification. (**a**) The peak current of different concentrations of PTH was verified *via* CV. The concentration of PTH was measured over a range of concentrations at 100 mV/s. (**b**) Calibration plot considering the peak current at 0.55 V for the detection of PTH. The error bars represent the standard deviation of three measurements. The inset depicts the linear range from 1 to 50 pg/mL.

The oxidation current for a PTH-spiked serum concentration ranging from 1 pg mL^−1^ to 10 ng mL^−1^, at a scan rate of 100 mV s^−1^, is shown in Fig. 4. The redox reaction was verified *via* CV, and the concentration of PTH was dependent on the oxidation peak at 0.55 eV (Fig. 4a). Each current peak was representative of a specific concentration of spiked serum. Moreover, the AP-MG sensor exhibited good reproducibility when the activity was measured three times (Fig. S5). As can be seen from Fig. 4a, the dashed line at 0.55 eV corresponds to the standard deviation (SD) of the peak current (n = 3), and the oxidation peak current increased with increasing PTH concentrations in the spiked serum samples. Figure 4b shows the calibration plot for PTH concentration. The linearity of the peak current ranges from 1 to 50 pg mL^−1^ (Fig. 4b, inset), and it was observed that the AP-MG sensor has good repeatability, with a relative standard deviation of 5.5%. It is observed that the sensor response in not linear throughout the PTH concentration range up to 10 ng mL^−1^. However, it is observed that the sensor response in linear in the PTH range of 1 to 50 pg mL^−1^, which is the actual requirement in the patient PTH monitoring^38^. We believe that the maximum detectable range of PTH is around 5 ng mL^−1^ which, in other words, because the presence of PTH in real serum sample is too small that the linearity in the low range is more important. Hence, our AP-MG sensor has a better detection range and lower detection limit than previously reported sensors^24^,^26^. A much greater peak current than usual was seen using this sensor. Nonetheless, the peak current was proportional to the concentration of PTH, indicating typical electrochemical behaviour for an immunoassay. We presume that this can be attributed to the use of MAbs, because a MAb recognizes a single epitope, enabling fine-scale detection and quantification of small differences in antigen. A polyclonal antibody is often used as the capture antibody, to pull down as much of the antigen as possible. Moreover, MG can enhance direct electron transfer between AP and the Au electrode. Additionally, an HRP-PTH-MoS_2_-Gr (HP-MG) sensor was used for analysis in the same concentration range, and it also produced results proportional to the concentration of PTH (Fig. S6). However, the slope of the calibration plot of the HP-MG sensor was much lower than that of the AP-MG sensor (Fig. S7), even though the results showed a similar trend to those obtained under conditions using IgG-conjugated enzymes. Finally, the AP-MG sensor was better at detecting PTH within the target concentration range. Therefore, we chose to use the AP-MG sensor to analyse the serum samples from patients with osteoporosis.

### Analysis of PTH in human serum

**Figure 5:**
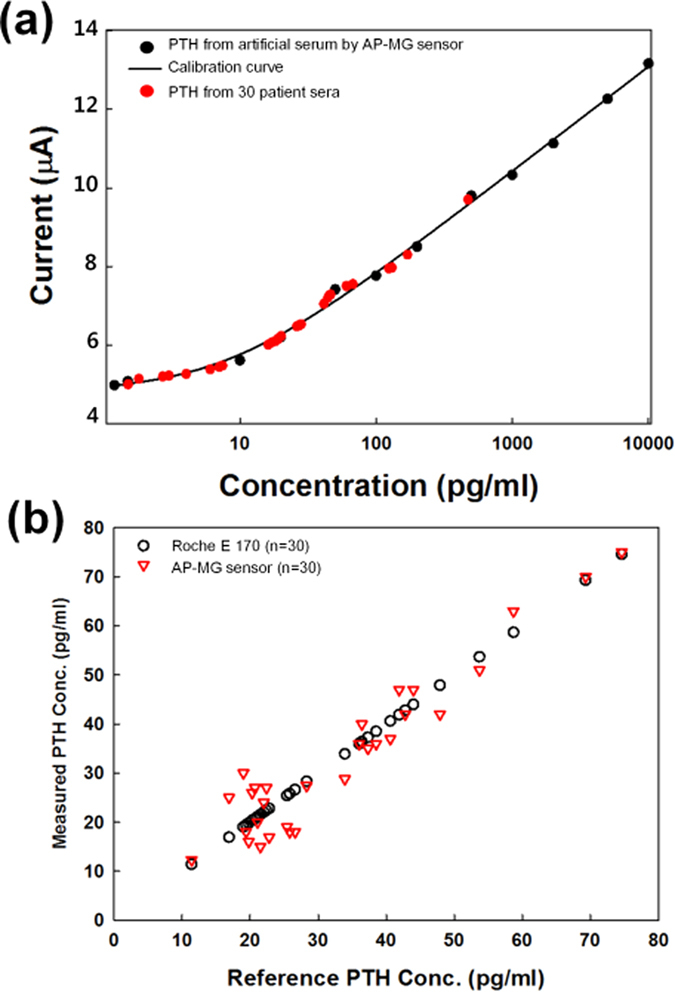
Performance of the AP-MG. (**a**) Performance using PTH from artificial sera, along with a standard calibration curve, and PTH from the sera of 30 patients. (**b**) A comparison of the PTH-detection performance of the AP-MG sensor with that of the Roche E 170, for serum samples from 30 patients.

The linear range, reproducibility, and repeatability of the AP-MG sensor was analysed. In this study, serum samples from patients with osteoporosis (n = 30) were analysed using the fabricated AP-MG sensor and an ‘E 170’ (E 170, Modular Analytics, Roche Diagnostics, Germany). The intact-PTH ECIA was used and aliquots were analysed accordingly. The peak current measured against PTH from artificial serum and PTH from patient serum was also analysed using the same AP-MG sensor (Fig. 5a). The peak current of PTH from patient serum samples was matched to the calibration plot generated from artificial serum samples. PTH was also evaluated in patient sera using an E 170 (Fig. 5b), and the PTH concentrations arrived using this approach were used as reference values. To analyse statistical differences two groups, an unpaired *t*-test with Welch’s correction was used. For all of our analyses, we considered a *P*-value below 0.01 to indicate statistical significance. The PTH concentrations measured using the AP-MG sensor showed a high correlation with those measured by E 170 (*R*^2^ > 0.95; *P* < 0.01). Moreover, both the reference PTH concentrations and those measured using the AP-MG sensor had a Pearson correlation coefficient of 0.95. We infer from this that the results obtained from the fabricated AP-MG sensor are in good agreement with those of the standard measurement device, E 170. This suggests the possibility of applying the AP-MG sensor to detect the concentration of PTH in human serum samples.

In summary, an MG composite was prepared using a hydrothermal method, assisted by l-cysteine, to modify an Au electrode, thereby preparing an electrochemical sensor. The modified MG electrode was used as sensing electrode for a sandwich enzyme-linked immunosorbent assay (ELISA), wherein the redox cycling permits detection of PTH. ALP and HRP were used with their respective substrates (AAP and HQ), because of their high electrocatalytic activity *via* hydrolysis. The AP-MG sensor performed well, with high sensitivity and reproducibility, when PTH concentrations in artificial serum samples were analysed by cyclic voltammetry within a linear range of 1–50 pg mL^−1^. Serum samples from patients with osteoporosis (n = 30) were analysed using the AP-MG sensor, based on the PTH calibration plot that had been generated using artificial serum samples. The calibration plots, generated using artificial serum samples and true samples from patients, were nearly identical. Moreover, the same patient samples were evaluated using an E 170, which uses an electrochemical immunoassay. Differences between the PTH concentrations measured using the AP-MG sensor, and those measured using the standard device, E 170, were statistically analysed using an unpaired *t*-test with Welch’s correction. This analysis revealed a high correlation with E 170 (*R*^2^ > 0.95; *P* < 0.01). The fabricated AP-MG sensor exhibited similar levels of accuracy and performance relative to E 170 for its ability to analyse PTH concentrations in serum samples from patients.

## Methods

### Reagents

Graphene oxide (GO), 1 mg mL^−1^, was synthesized in our laboratory^39^. Na_2_MoO_4_ · H_2_O, l-cysteine, and phosphate-buffered saline (PBS; pH 7.0) were purchased from Sigma Aldrich (P4417, USA). Horseradish peroxidase (HRP)-conjugated goat anti-mouse IgG (NC11430KR), and Blocker^TM^ BSA (10X) in PBS, were purchased from Thermo-Scientific. Alkaline phosphatase (ALP)-conjugated goat anti-mouse IgG (A3562), hydroquinone (HQ, H3660), hydrogen peroxide solution (216763), and (3-aminopropyl)triethoxysilane (APTES) were purchased from Sigma Aldrich. Ascorbic acid-2-phosphate (AAP) magnesium salt hydrate was obtained from Wako Pure Chemical Industries, Ltd (Product No. 013-12061, Osaka, Japan). Monoclonal PTH antibody, PTH antigen, and polyclonal PTH antibody were all sourced from Fitzgerald Industries. Deionized water (DIW, >18 MΩ cm), used to prepare all solutions in this study, was obtained from a water purification system.

### Instrumentation

The structure of the MG composite was characterized by scanning electron microscopy (SEM, JEOL, JSM-6700F, Japan), high-resolution transmission electron microscopy (HR-TEM, JEOL, JEM-ARM200F, Japan), Fourier transform infrared spectrometry (FT-IR, Bruker, TENSOR27, Germany), and X-ray diffraction (XRD, Bruker, D8 ADVANCE, Germany). For Raman spectroscopy (Alpha300 M+, WITec GmbH), the wavelength and power of the laser were 532 nm and 2 mW, respectively. X-ray photoelectron spectroscopy (ESCA2000, VG Microtech Inc.) was employed to determine the chemical composition of materials. The zeta potential of the aqueous dispersion of MG was evaluated using an electrophoretic light-scattering photometer (ELS-Z, Otsuka, Japan). Hydrophilic treatment was performed using an oxygen plasma in a CUTE chamber (Femto Science, Republic of Korea). Electrochemical immunoassay measurements were performed on a VersaSTAT 3 potentiostat/galvanostat (Ametek, Princeton Applied Research, USA) with a custom-made Au-printed circuit board (PCB) sensor^40^.

### MG synthesis and sensor fabrication

GO was synthesized from natural graphite using the method of Hummers and Offeman, with minor modifications^39^. Briefly, the MG composite was prepared in solution and modified by replacing NH_2_CSNH_2_^41^ with l-cysteine^42^. Five millilitres of GO (1 mg mL^−1^) was mixed with Na_2_MoO_4_ · H_2_O (0.1 g). After sonication and stirring for 30 min, the pH of the solution was adjusted to 7.0 with 0.1 M NaOH. A sample of l-cysteine (0.2 g) was dissolved in 2.5 mL of double-distilled water and placed into a 50 mL Teflon-lined stainless steel autoclave. The container was sealed and heated at 180 °C for 36 h. After cooling in air, the black precipitate from each composite sample was collected by centrifugation (12,000 rpm for 20 min) and washed separately with double-distilled water and ethanol. The sample was dried in a vacuum oven at 80 °C for 12 h.

The MG composite powder (2.0 mg) was weighed and dispersed in 2 mL DIW by ultra-sonication for 1 h. Both MG and APTES (3% in ethanol) were added to a vial containing anhydrous ethanol, and maintained at room temperature for 10 h, with magnetic stirring (800 rpm). Following the reaction, the solution containing MG was separated by centrifugation, washed twice with ethanol, and used thereafter for further characterization. The customized Au-PCB (d = 2 mm) sensor employed in the present experiment was surface-cleaned by isopropanol and rinsed thoroughly with DIW. Before performing any surface modification, the Au-PCB working surface was treated with oxygen plasma for 2 min^40^. The Au electrode was pretreated with O_2_ plasma at a base pressure of 5 × 10^−2^ Torr, a working pressure of 7.8 × 10^−1^ Torr, 50 watts power, and an oxygen flow rate of 45 sccm for 2 min. The aqueous suspension of MG, which had been prepared after centrifugation, was drop-coated in 10 μL on the Au electrode and left to evaporate at room temperature for 1 h. This modified MG electrode was now ready as a sensing electrode for sandwich ELISA. In an electrochemical ELISA, signals depend on the surface concentration of an enzymatic product at a sensing electrode. After fabrication of MG on the Au electrode, which will hence forth be referred to as the MG sensor, the sensor was thoroughly rinsed with DIW, twice. Then, the MG sensor was modified by immersion into a 10 μL of 1/1,000 diluted IgG solution, for 1 h at 4 °C. A solution containing IgG molecules bearing a separately conjugated enzyme (ALP/HRP) was used to attach the enzyme to the MG sensor. Henceforth, the enzyme-modified MG sensors will be referred to as either the AI-MG or the HI-MG sensor. An electrochemical measurement cell was assembled with the resulting electrodes and 6 mL of a Tris buffer solution containing ALP substrate (1 mM AAP) was injected into the cell. An enzymatic reaction occurred and the electrochemical measurement was performed using a VersaSTAT. The electrochemical cell included an Au electrode, an Ag/AgCl electrode (saturated 3 M KCl), and a platinum (Pt) electrode as the working, reference, and auxiliary electrodes, respectively (Fig. S8).

**Figure 6:**
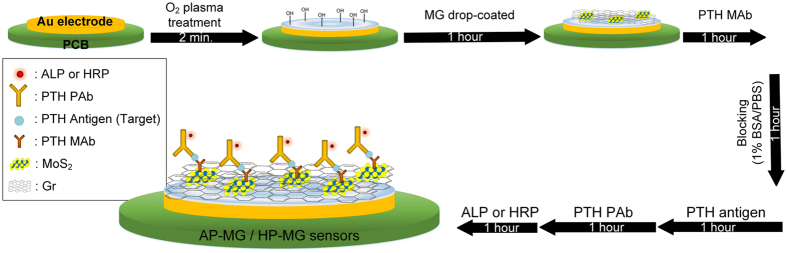
Schematic for the fabrication of AP-MG and HP-MG sensors for the detection of PTH from artificial and patient-derived sera.

After successful evaluation of sensing mechanism with the AI-MG and HI-MG sensors, further study were carried out. AP-MG and HP-MG sensors were fabricated and the fabrication process is as shown in Fig. 6. The MG electrode was prepared using the previously described steps and was thoroughly rinsed with DIW, twice. The MG electrode was covered at 4 °C for 1 h with 10 μL of a 200 μg mL^−1^ solution of a MAb against PTH as described in Fig. 6. Physically absorbed PTH molecules were removed by rinsing with buffer. PBSB solution blocked the PTH MAb on the MG electrode at 4 °C for 1 h. Following aspiration, the electrode was incubated with a 10 μL/drop concentration of PTH antigen for 1 h at 4 °C and washed with rinsing buffer (Fig. 6). The PTH antigen to be measured must contain at least two antigenic sites capable of binding an antibody because at least two antibodies act in the sandwich ELISA. Either MAbs or polyclonal antibodies (PAbs) can be used for capture and detection in sandwich ELISA systems. Binding of PTH MAb and PTH antigen was achieved; then, the MG electrode was immersed in a 10 μL/drop of PTH PAb solution (1/100 diluted) for 1 h at 4 °C. In general there is effect of pH in tested solution on the sensing performance, hence we perform experiments for AAP solution in 50 mM Tris-HCl + 10 mM MgCl_2_ in the range of 3 to 12 pH (Fig. S8).

### Serum samples from patients

Serum samples were obtained from 30 patients with osteoporosis at Seoul National University Bundang Hospital (SNUBH; Bundang, Republic of Korea). These patients were female between 50–60 years and they were in menopause. Serum samples from these patients were used to investigate PTH levels using the fabricated AP-MG sensor, and the results were compared with those obtained using a standard electrochemical immunoassay (ECIA) device (E 170, Modular Analytics, Roche Diagnostics, Germany). Prior to commencing the study, the study protocol, amendments, and informed consent procedures were approved by the Institutional Review Board and the research ethics committee of SNUBH. The study was conducted in compliance with the protocol, the current revision of the Declaration of Helsinki, Good Clinical Practice, the applicable regulatory requirements, and all the relevant guidelines of the International Conference for Harmonisation. Before exposure to any study-related procedures, all patients gave their written informed consent. Patients were informed by the investigator about the aims, methods, anticipated benefits, and potential hazards of participating in the study.

## Additional Information

**How to cite this article**: Kim, H.-U. *et al*. A sensitive electrochemical sensor for *in vitro* detection of parathyroid hormone based on MoS_2_-graphene composite. *Sci. Rep.* **6**, 34587; doi: 10.1038/srep34587 (2016).

## Supplementary Material

Supplementary Information
