# Supplementary material for: A sensitive electrochemical sensor for in vitro detection of parathyroid hormone based on a MoS2-graphene composite
Source: Sci Rep. 2016 Oct 3;6:34587. doi: 10.1038/srep34587 (PMC5046135; doi:10.1038/srep34587)
Supplement: Supplementary Information [file srep34587-s1.pdf]

A sensitive electrochemical sensor for *in vitro* detection of parathyroid hormone based on MoS_2-graphene composite

Hyeong-U Kim[1,†], Hye Youn Kim[2,†], Atul Kulkarni[3], Chisung Ahn[1], Yinhua Jin[1], Yeongseok Kim[3], Kook-Nyung Lee[2], Min-Ho Lee[2,*] and Taesung Kim[1,3,*]

[1] SKKU Advanced Institute of Nano Technology (SAINT), Sungkyunkwan University, Suwon, Gyeonggi-do, South Korea

[2] Korea Electronics Technology Institute, Seongnam, Gyeonggi-do, South Korea

[3] Mechanical Engineering, Sungkyunkwan University, Suwon, Gyeonggi-do, South Korea

[†] These authors contributed equally to this work

Corresponding author: Taesung Kim, Min-Ho Lee

E-mail: tkim@skku.edu, mhlee@keti.re.kr

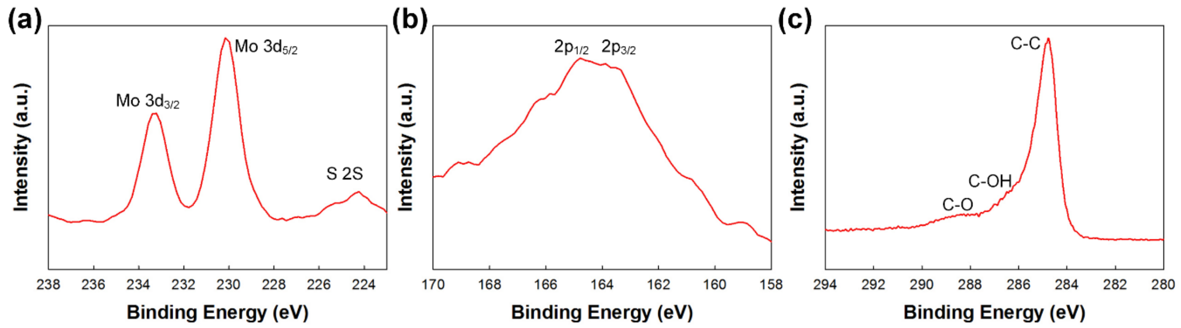

Supplementary Fig. S1 High resolution XPS spectra corresponding to MG composite (a) Mo 3d, S 2p (b) 2p and (c) C 1s

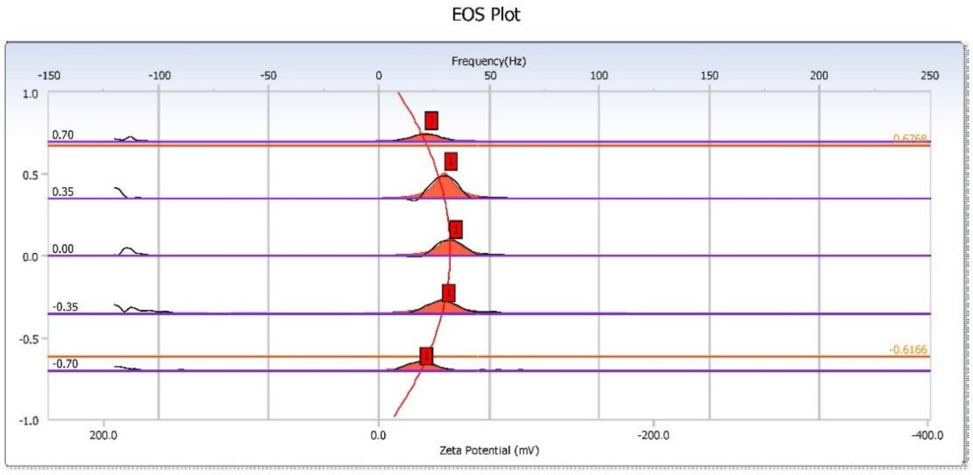

Measurement Results

Zeta Potential	: -35.25	(mV)	Doppler shift	: 21.96	(Hz)
Mobility	: -2.749e-004	(cm²/Vs)	Base Frequency	: 120.2	(Hz)
Conductivity	: 0.0636	(mS/cm)	Conversion Equation	: Smoluchowski	
Zeta Potential of Cell			Diluent Properties		
Upper Surface	: -20.98	(mV)	Diluent Name	: WATER	
Lower Surface	: -25.82	(mV)	Temperature	: 25.0	(°C)
Cell Condition			Refractive Index	: 1.3328	
Cell Type	: Flow Cell		Viscosity	: 0.8878	(cP)
Avg. Electric Field	: -16.54	(V/cm)	Dielectric Constant	: 78.3	
Avg. Current	: -0.05	(mA)			

Supplementary Fig. S2 Zeta potential of MG composite

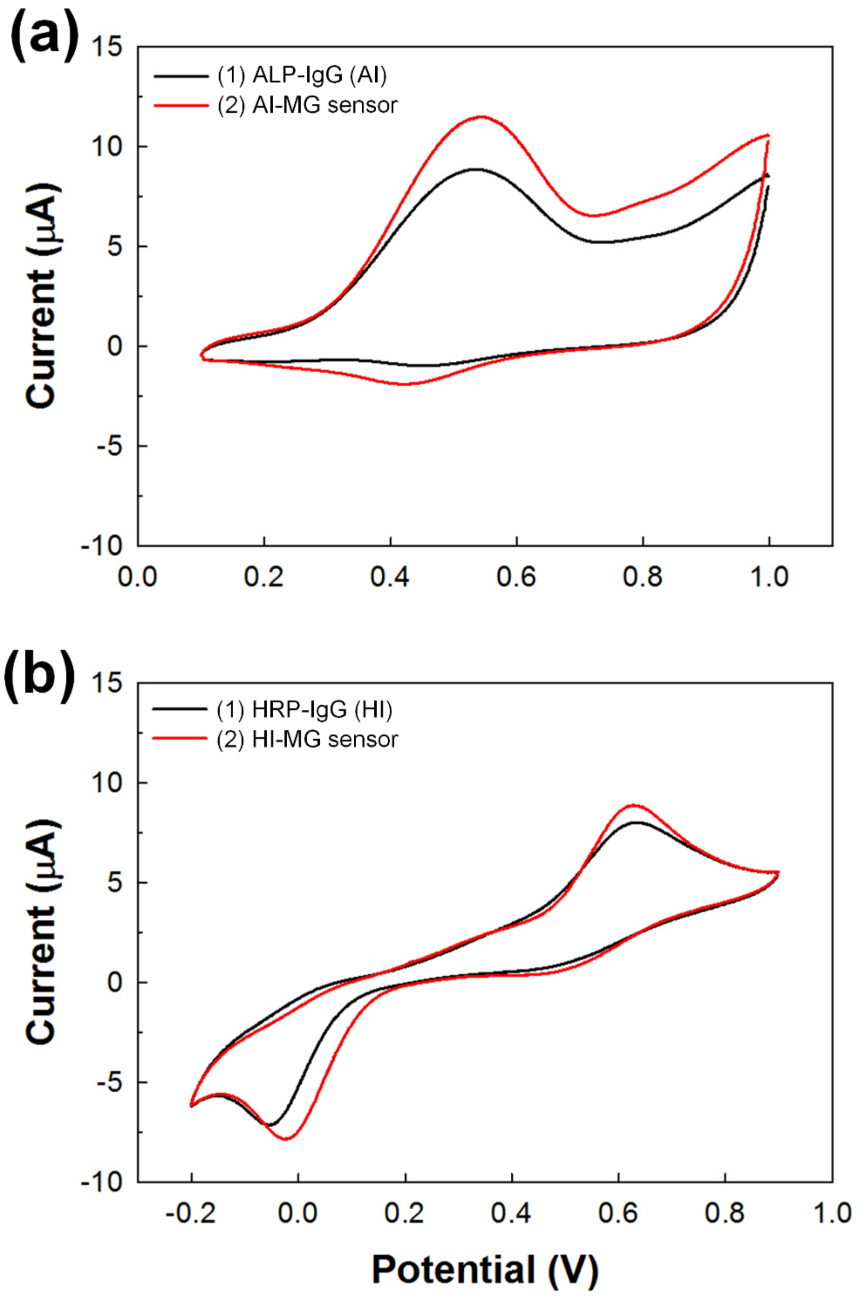

Supplementary Fig. S3 Cyclic voltammogram (CV) measurement of modified electrodes (a) (1) AI electrode (2) AI-MG electrode at scan rate 100 mV/s (b) (1) HI electrode (2) HI-MG electrode at scan rate 100 mV/s, respectively

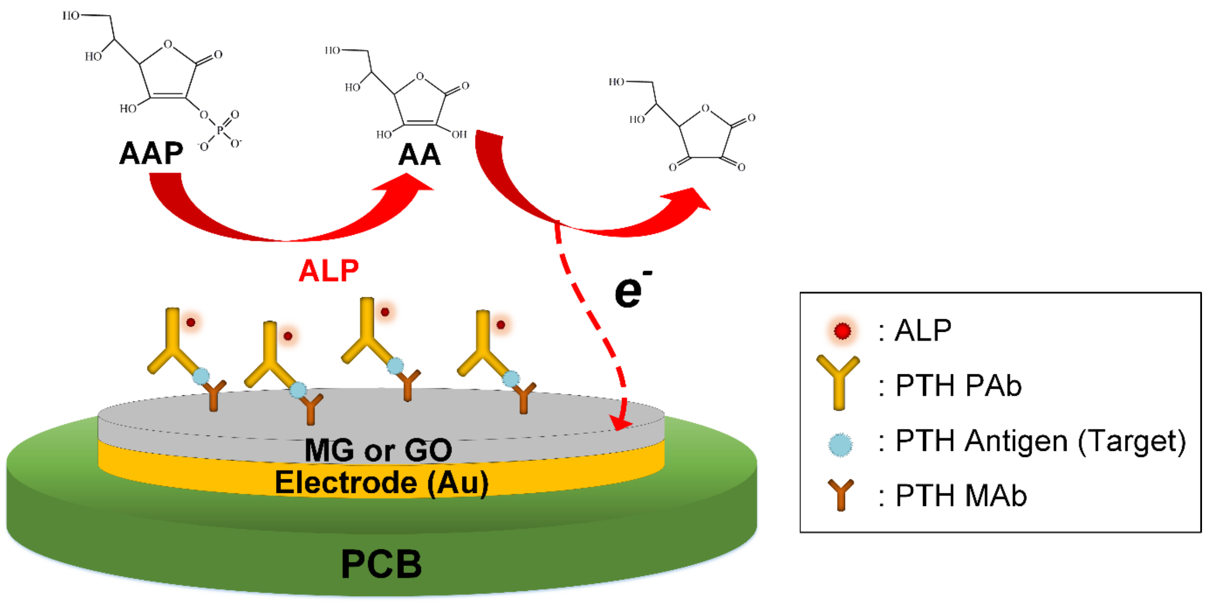

Supplementary Fig. S4 Mechanism of AP-GO and AP-MG sensors with AAP substrate

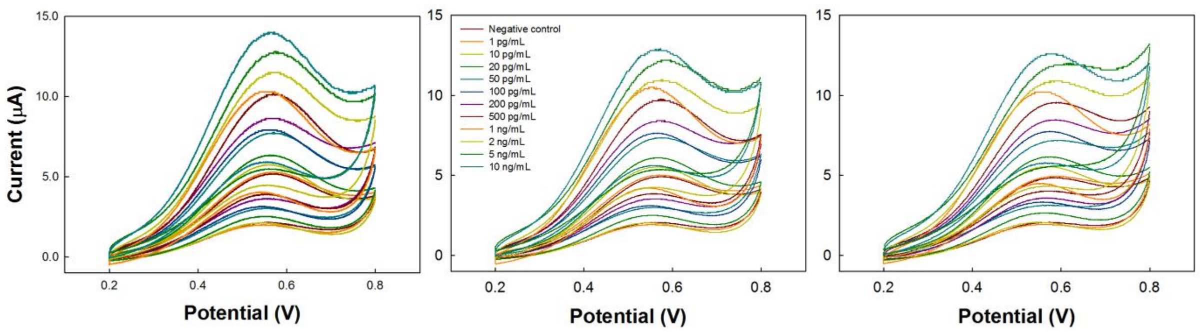

Supplementary Fig. S5 Three cycling of AP-MG sensor with different concentration of PTH

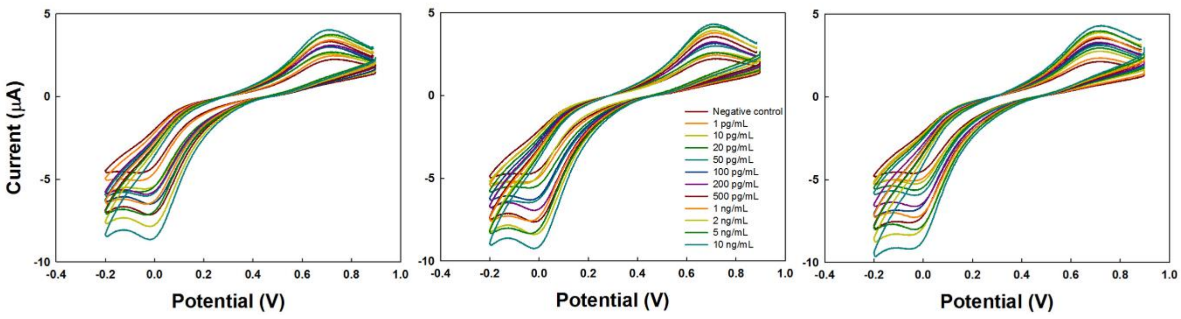

Supplementary Fig. S6 Three cycling of HP-MG sensor with different concentration of PTH

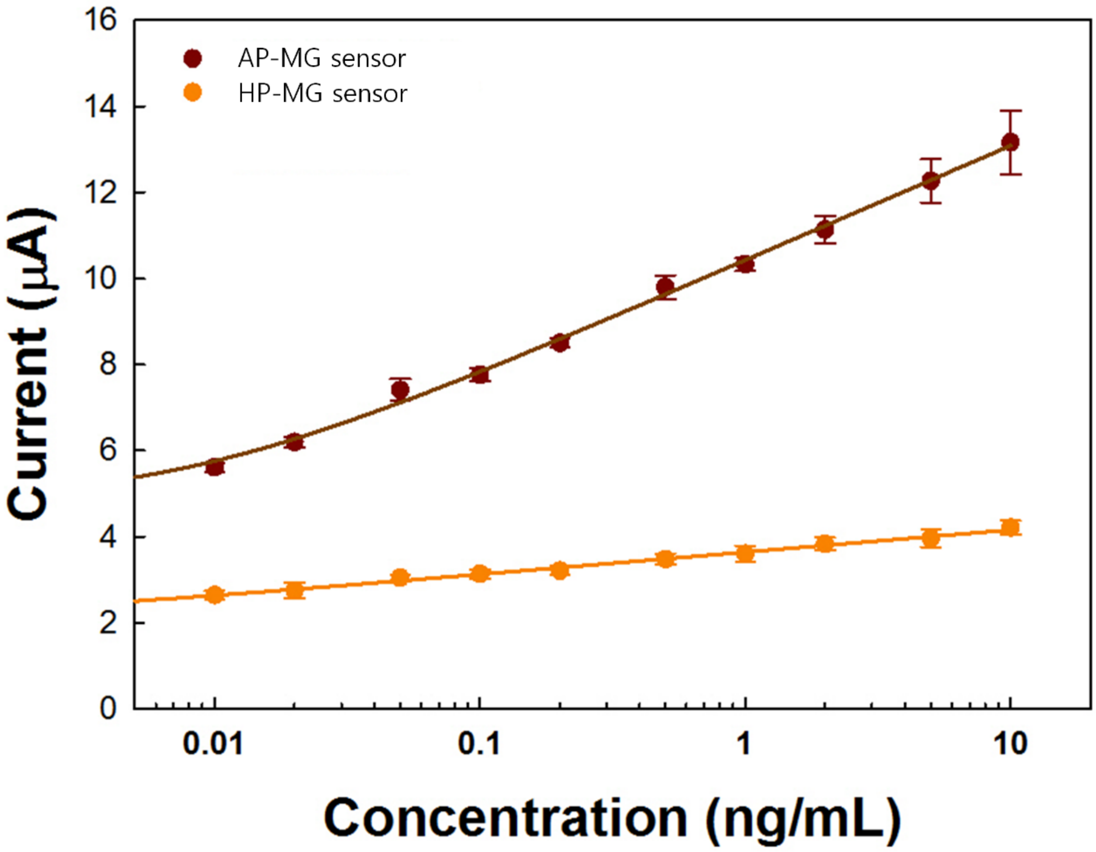

Supplementary Fig. S7 PTH calibration curve obtained at PTH concentration ranging from 1 pg/mL to 10 ng/mL by both AP-MG and HP-MG sensors

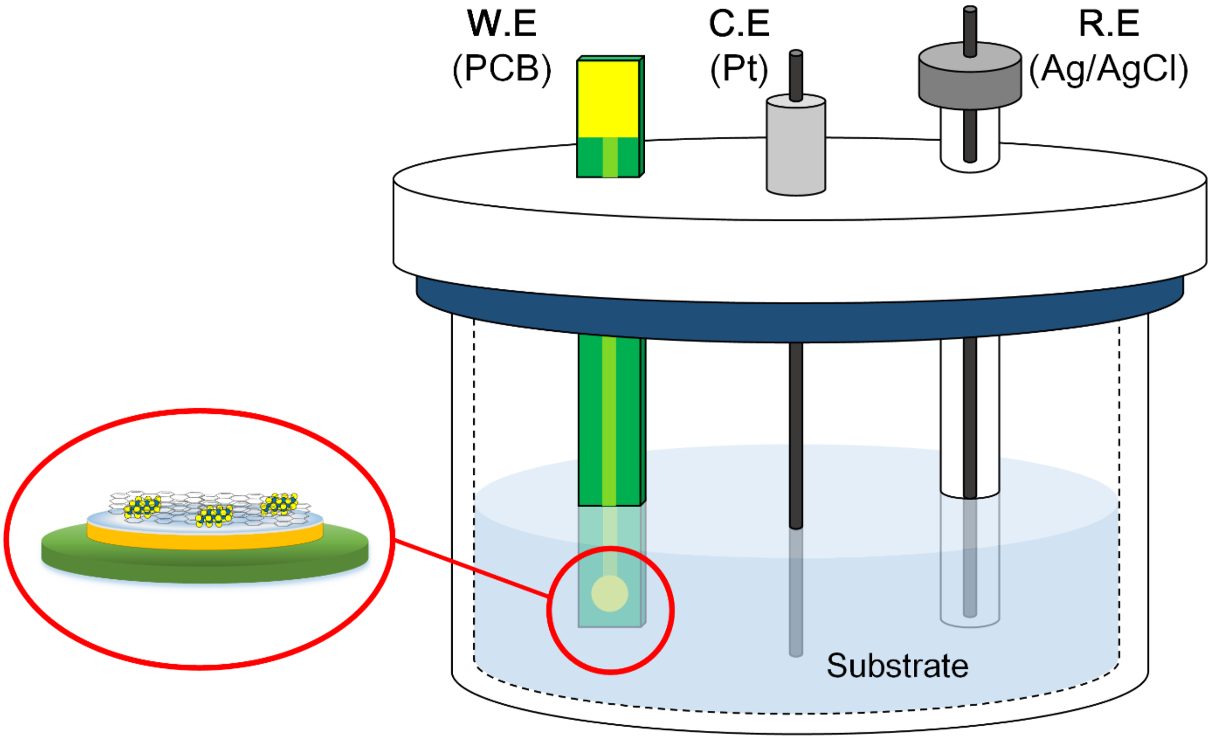

Supplementary Fig. S8 Schematic representation of experimental setup wherein Au-PCB as working electrode, Pt as counter electrode and Ag/AgCl (Sat'd 3M KCl) as reference electr ode, respectively. MG is deposited on Au-PCB electrode

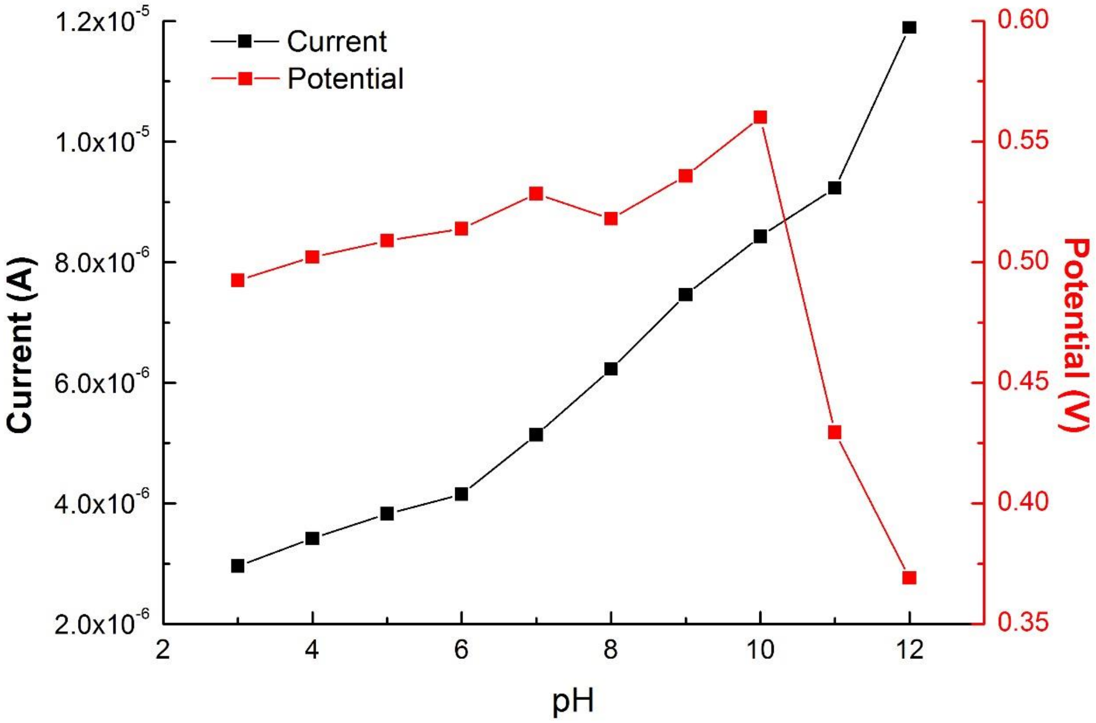

Supplementary Fig. S9 Effect of (AAP solution in 50 mM Tris-HCl + 10 mM $MgCl_2$) pH on AP-MG sensor. The maximum potential (0.55 V) is observed at pH 9.6
